# Supplementary material for: Actin-Dependent Mechanism of Tumor Progression Induced by a Dysfunction of p53 Tumor Suppressor
Source: Cancers (Basel). 2024 Mar 11;16(6):1123. doi: 10.3390/cancers16061123 (PMC10969470; doi:10.3390/cancers16061123)
Supplement: Supplementary file 1 [file cancers-16-01123-s001.zip › cancers-2897960 supplementary.pdf]

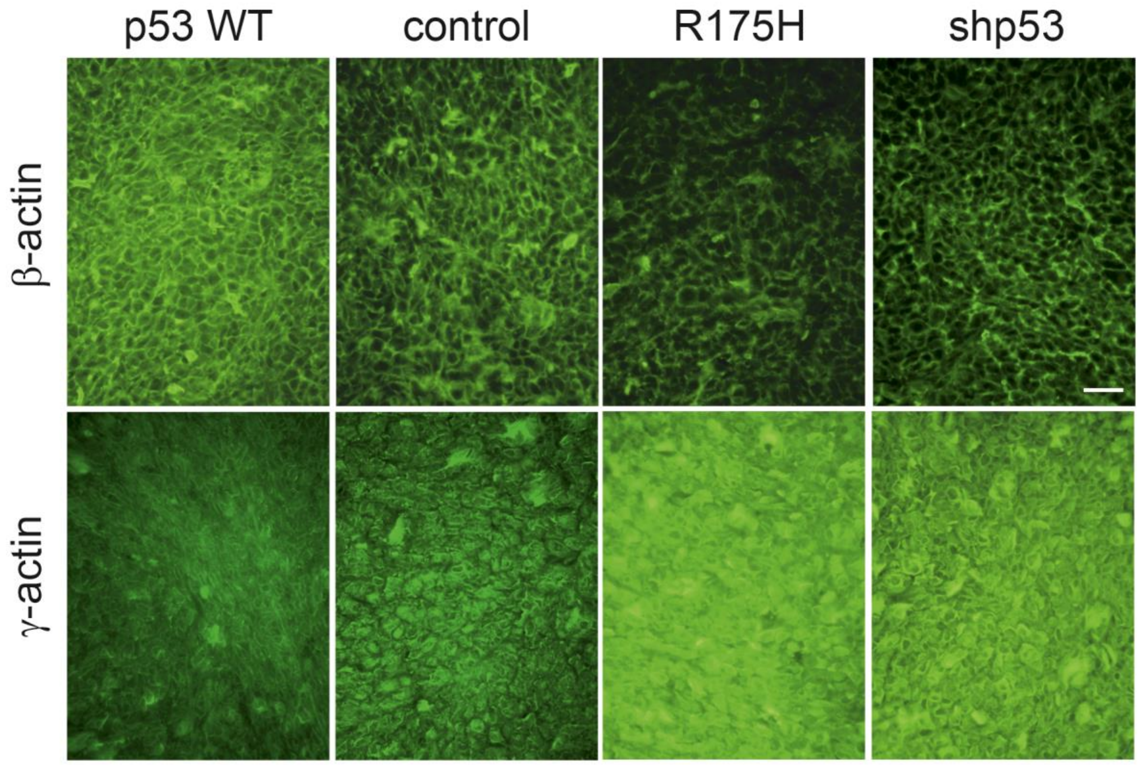

Figure S1. Fluorescent IHC staining for β-actin (upper panel) and γ-actin (lower panel) of H116 subcutaneous xenografts with various p53 status. Scale bar represents 50 μm.

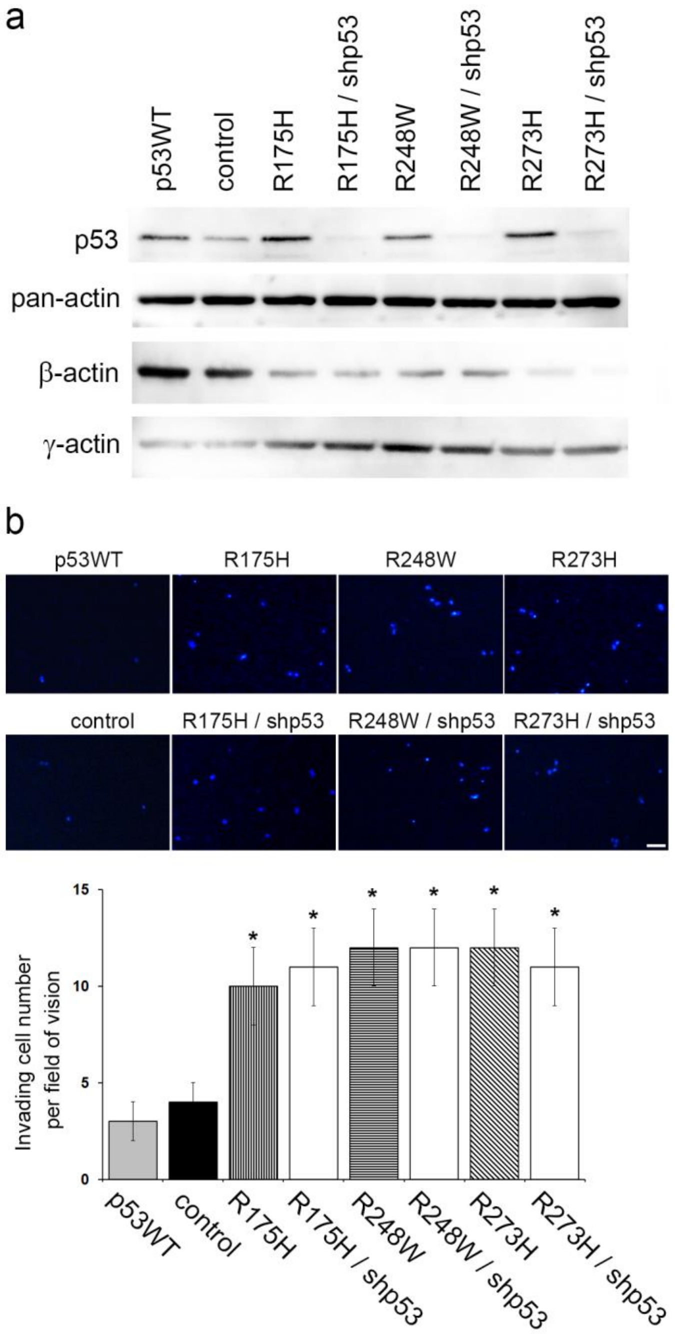

Figure S2. Analysis of shRNA-induced down-regulation of exogenous expression of p53 mutants in A549 cells of actin isoform balance and invasion activity. a. WB analysis of A549 cells with various p53 statuses. Representative images are shown. b. Invasive activity of A549 cells through matrigel-coated membranes. Typical fields of vision are presented, DAPI staining, scale bar represents 50 μm (upper panel). Graphs represent mean ± SD (lower panel).

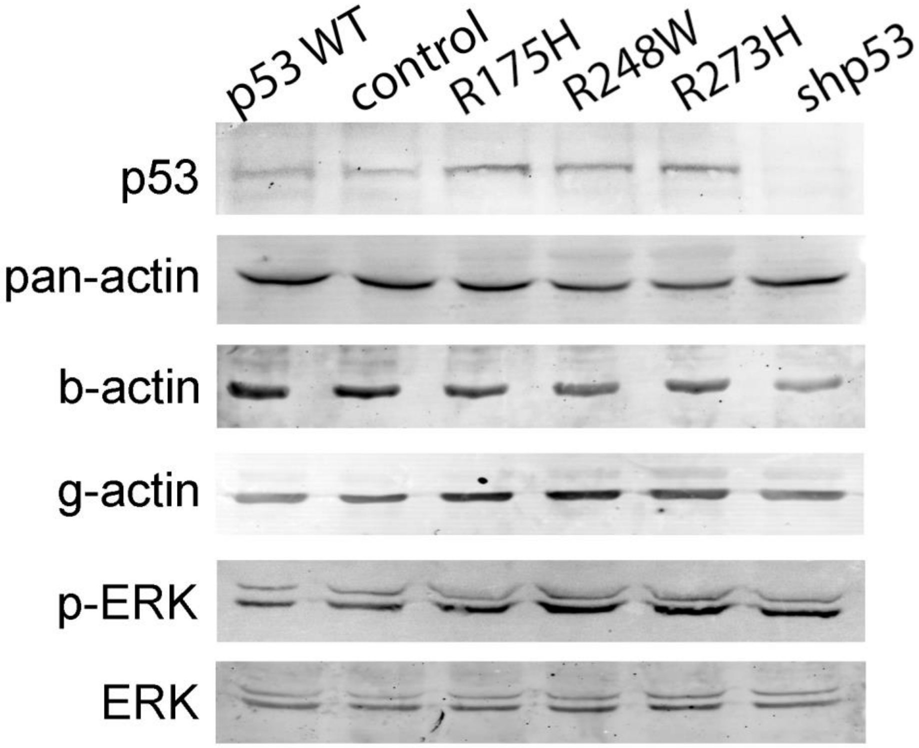

Figure S3. WB analysis of HCT116 cells with various p53 status.
